# The Bactericidal Effect of Dendritic Copper Microparticles, Contained in an Alginate Matrix, on *Escherichia coli*

**DOI:** 10.1371/journal.pone.0096225

**Published:** 2014-05-15

**Authors:** Simon F. Thomas, Paul Rooks, Fabian Rudin, Sov Atkinson, Paul Goddard, Rachel Bransgrove, Paul T. Mason, Michael J. Allen

**Affiliations:** 1 Plymouth Martine Laboratory Applications Ltd, Plymouth, United Kingdom; 2 Plymouth Marine Laboratory, Plymouth, United Kingdom; 3 Protein Technologies Ltd, Manchester, United Kingdom; National Central University, Taiwan

## Abstract

Although the bactericidal effect of copper has been known for centuries, there is a current resurgence of interest in the use of this element as an antimicrobial agent. During this study the use of dendritic copper microparticles embedded in an alginate matrix as a rapid method for the deactivation of *Escherichia coli* ATCC 11775 was investigated. The copper/alginate produced a decrease in the minimum inhibitory concentration from free copper powder dispersed in the media from 0.25 to 0.065 mg/ml. Beads loaded with 4% Cu deactivated 99.97% of bacteria after 90 minutes, compared to a 44.2% reduction in viability in the equivalent free copper powder treatment. There was no observed loss in the efficacy of this method with increasing bacterial loading up to 10^6^ cells/ml, however only 88.2% of *E. coli* were deactivated after 90 minutes at a loading of 10^8^ cells/ml. The efficacy of this method was highly dependent on the oxygen content of the media, with a 4.01% increase in viable bacteria observed under anoxic conditions compared to a >99% reduction in bacterial viability in oxygen tensions above 50% of saturation. Scanning electron micrographs (SEM) of the beads indicated that the dendritic copper particles sit as discrete clusters within a layered alginate matrix, and that the external surface of the beads has a scale-like appearance with dendritic copper particles extruding. *E. coli* cells visualised using SEM indicated a loss of cellular integrity upon Cu bead treatment with obvious visible blebbing. This study indicates the use of microscale dendritic particles of Cu embedded in an alginate matrix to effectively deactivate *E. coli* cells and opens the possibility of their application within effective water treatment processes, especially in high particulate waste streams where conventional methods, such as UV treatment or chlorination, are ineffective or inappropriate.

## Introduction

Elemental copper has been used as an anti-spoiling agent and preservative for over two millennia, predating the discovery of the role of microbes in disease during the late nineteenth century [1]. The use of copper as an antimicrobial agent has recently provoked renewed interest, due to its rapid and extensive activity, especially on dry surfaces. For example, *Salmonella enterica* and *Campylobacter jejuni* are destroyed within two hours on a dry copper surface [2] and Santo *et al* showed that *Staphlococcus haemolyticus* was deactivated within minutes, accompanied by extensive membrane damage [3]. This has resulted in the increasing popularity of copper surfaces in hospital and institutional environments as an active antimicrobial surface, on items such as door knobs and bench tops. The anti-microbial mechanism of copper in such applications is poorly characterised, although recent work suggests that toxicity is due to the oxidation of the surface layer and consequent donation of electrons to cell wall components, such as lipids and proteins, resulting in a rapid reduction of cell integrity [3]. Other mechanisms such as internal protein binding and DNA mutation have also been suggested [4], [5] and indeed *in vitro* experimentation has showed that Cu can bind to DNA. However, Macomber *et al* indicated that *in vivo*, Cu^2+^ ions were sequestered by the glutathione naturally produced by *E. coli* thus preventing oxidative damage to DNA through Fenton-like reactions [6].

Unfortunately, the use of free Cu salts as a water sterilisation method is restricted by toxicity to both aquatic organisms and humans, although a combination of Cu with Ag is often used to inhibit *Legionella* sp. in cooling tower water [7]. Accumulation of Cu in the gills of freshwater fish has been shown to inhibit Na^+^ influx and reduce Na-K ATPase activity, and just 1.2 µg/l Cu^2+^ has been shown to produce chronic toxicity for the freshwater mussel, *Lamsilis siliquoidea* [8] and 37 µg/l Cu^2+^ is toxic to the Minnesota Sculpin [9]. The use of elemental copper is therefore preferential, but surface coating of large vessels with copper results in a slower antimicrobial action due to reduced contact time. Micro and nano copper particles offered increased relative surface areas, but are difficult to evenly distribute in water and the entrapment of these particles in a matrix is desirable. Alginate is obtained from a variety of brown macroalgae, such as *Laminaria* sp. and *Macrocystis pyrifera*, and is widely used as an emulsifier, thickener and as a matrix for the binding of proteins, enzymes and metals [10], [11]. It is an anionic polysaccharide consisting of homopolymeric blocks of (1–4)-linked β-D-mannuronate and its C-5 epimer α-L-guluronate residues, covalently linked together in different sequences or blocks [12]. Alginate complexes with metal ions via its carboxylate groups, and alginate binding with Cu salts and subsequent antimicrobial activity has been shown previously [13].

In the following article, the use of dendritic copper microparticles bound within an alginate matrix as an antimicrobial agent is assessed and the effect of a key environmental parameter, dissolved oxygen content, on the antimicrobial activity ascertained.

## Methods

### Bead Formation

Beads were prepared according to a modification of the method described by Albarghouthi *et al* [14]. Briefly 4% (w:v) sodium alginate (Sigma Aldrich, Poole, UK) was dissolved in deionised water by heating to 60°C whilst stirring on a magnetic stirrer at 100 rpm. Once dissolved, the alginate solution was autoclaved at 15 psi for 15 minutes. Once cooled, dendritic copper microparticles (Specific surface 1600 cm^2^/g, <63 µm) were added where appropriate at a concentration between 1–4%. The particles were dispersed by sonication using a VibraCell sonic probe (Sonics and materials, Dansbury, Ct, USA) for 60 minutes. The mixture was stored at 4°C until further processing.

Beads were formed by the drop wise addition of alginate/copper mixture via a 0.8 mm gauge needle into a 4°C solution of CaCl_2_ (2.5% w:v). Once formed, beads were washed in deionised water three times and stored in deionised water at 4°C until used.

### Growth of *E. coli* ATCC 11775

*E. coli* ATCC 11775 from −80°C stored stocks was inoculated into 50 ml of sterilised Luria-Bertani (LB) broth (Sigma Aldrich, Poole, UK) and incubated overnight at 37°C in a shaking incubator (Bibby scientific, Staffordshire, UK) at 150 rpm; 1 ml of this stock was then added to 250 ml of LB broth, which was incubated overnight under the same conditions. The bacterial cell numbers were then determined using the Gram staining method [15] and subsequent enumeration using an improved Neubauer hemocytometer.

### Antimicrobial Assay Procedure

All assays were performed in a media containing 0.5 g/l Bacto peptone and 0.2 g/l Yeast extract (both Sigma Aldrich, Poole, UK). Briefly, *E. coli* was added at the appropriate concentration, to 50 ml of assay media, and then shaken at 37°C in a shaking incubator (Bibby scientific, Staffordshire, UK) at 150 rpm for 30 minutes to allow acclimation. Alginate beads (1 g) were then aseptically added giving a final Cu copper concentration of between 0.125 and 1 mg/ml. The samples returned to the shaking incubator, and 1 ml of sample was then removed at 15 minute intervals for microbial examination. Each sample was serial diluted at 4°C in 1/8 strength Ringers solution (Fisher, Leicester, UK) and plated onto 90 mm Petri dishes containing MacConkey media number 2 (Sigma Aldrich, Poole, UK). The Petri dishes were then incubated overnight at 37°C and the distinctive pink colonies (indicative of *E. coli*) counted. All treatments were prepared in triplicate, and technical triplicate replicates also performed. Minimum inhibitory concentrations (MIC) of Cu to *E. coli* were determined using the methods described by Wiegand *et al* [16]. Free copper was dispersed into the media prior to bacterial inoculation by sonication.

The effects of dissolved oxygen (DO) on copper-induced microbial toxicity were assessed using the same methods as above, but at a 2.5 litre scale in an Infors Minifor fermenter (Reigate, UK) and DO was measured via the manufacturer’s proprietary probe and adjusted by air injection and variable stirring rate via the equipment’s software.

### Scanning Electron Microscopy (SEM)

Samples were prepared for analysis by sputter coating with chrome (for bacteria) and carbon (for beads and copper identification), using an EMTECH K450X sputter coater (Wrexham, UK). Prepared samples were subsequently analysed on a JEOL JSM-700F field emission scanning electron microscope (JEOL, Welwyn Garden City, UK).

### Quantification of Cu^2+^ Leaching and Absorption by Alginate Beads

Cu-Alginate beads (5 g) were placed in a 250 cm^3^ Schott bottle with 50 cm^3^ of MilliQ water. Lids were screwed on to prevent additional air from entering the system. 100 µl aliquots of each sample were taken at 0, 1, 2, 4, 8, 12, 24, 36, 48 hours and placed in Eppendorfs for [Cu^2+^] analysis. Analysis of [Cu^2+^] in MilliQ water used in all the samples and MilliQ water with 5 g beads without copper was performed, to account for any Cu^2+^ from beads and water. These readings were subsequently deducted from those of the samples.

Absorption of Cu^2+^ by alginate beads was determined by measuring the free copper concentration over time using a Palintest 7500 photometer (Gateshead, UK) with the proprietary free copper test kit, whilst using the manufacturer’s instructions.

## Results

### Copper/Alginate Bead Complex as an Antimicrobial Agent

**Figure 1:**
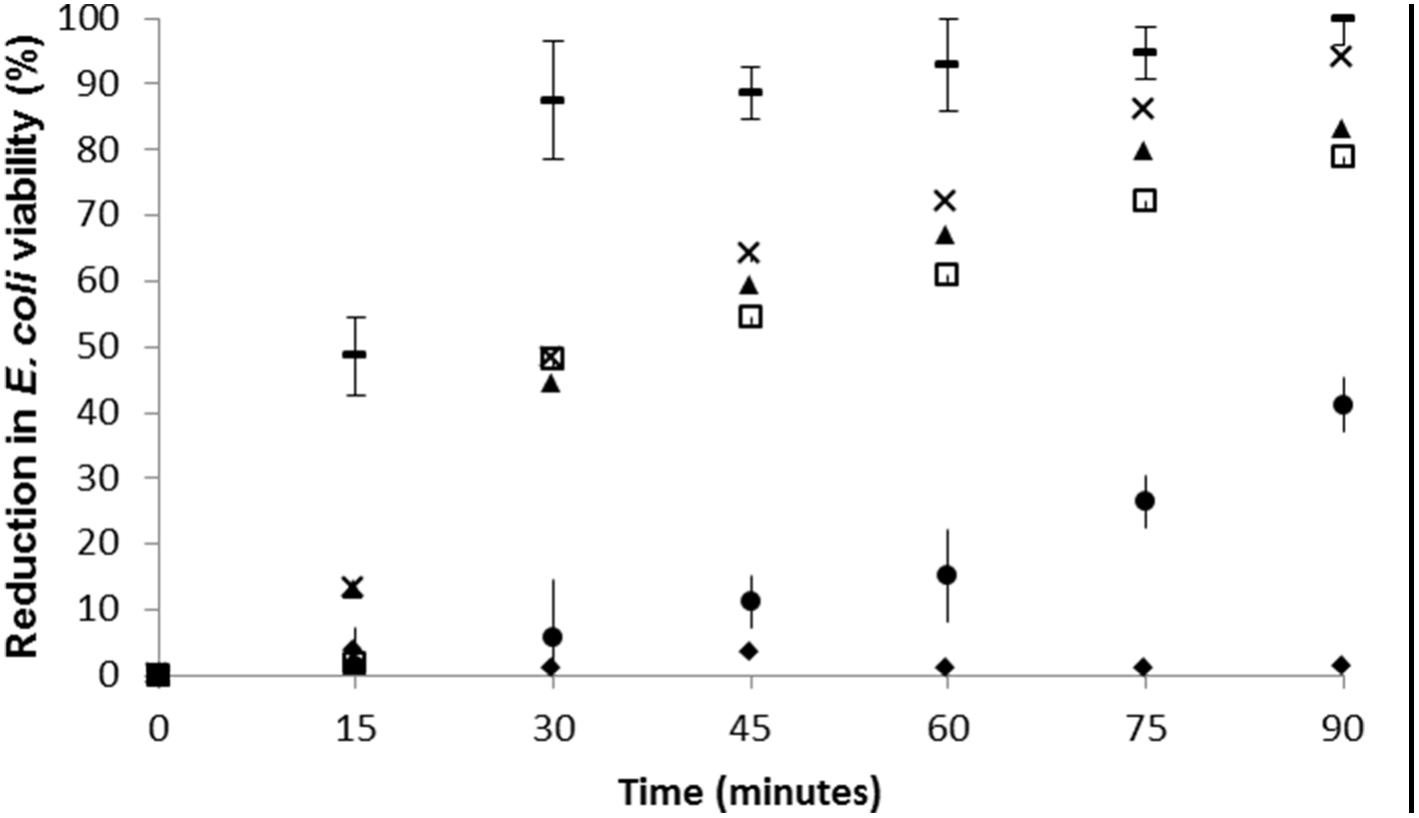
The biocidal effect of differing Cu concentrations entrapped in alginate beads on *E. coli* ATCC 11775 over time. Black diamond = Plain alginate beads. White square = 1% Cu W:W. Black triangle = 2% Cu. Black cross = 3% Cu, Black dash = 4% Cu. Black circle = 4% copper powder not associated with alginate. Bars denote standard errors.

**Figure 2:**
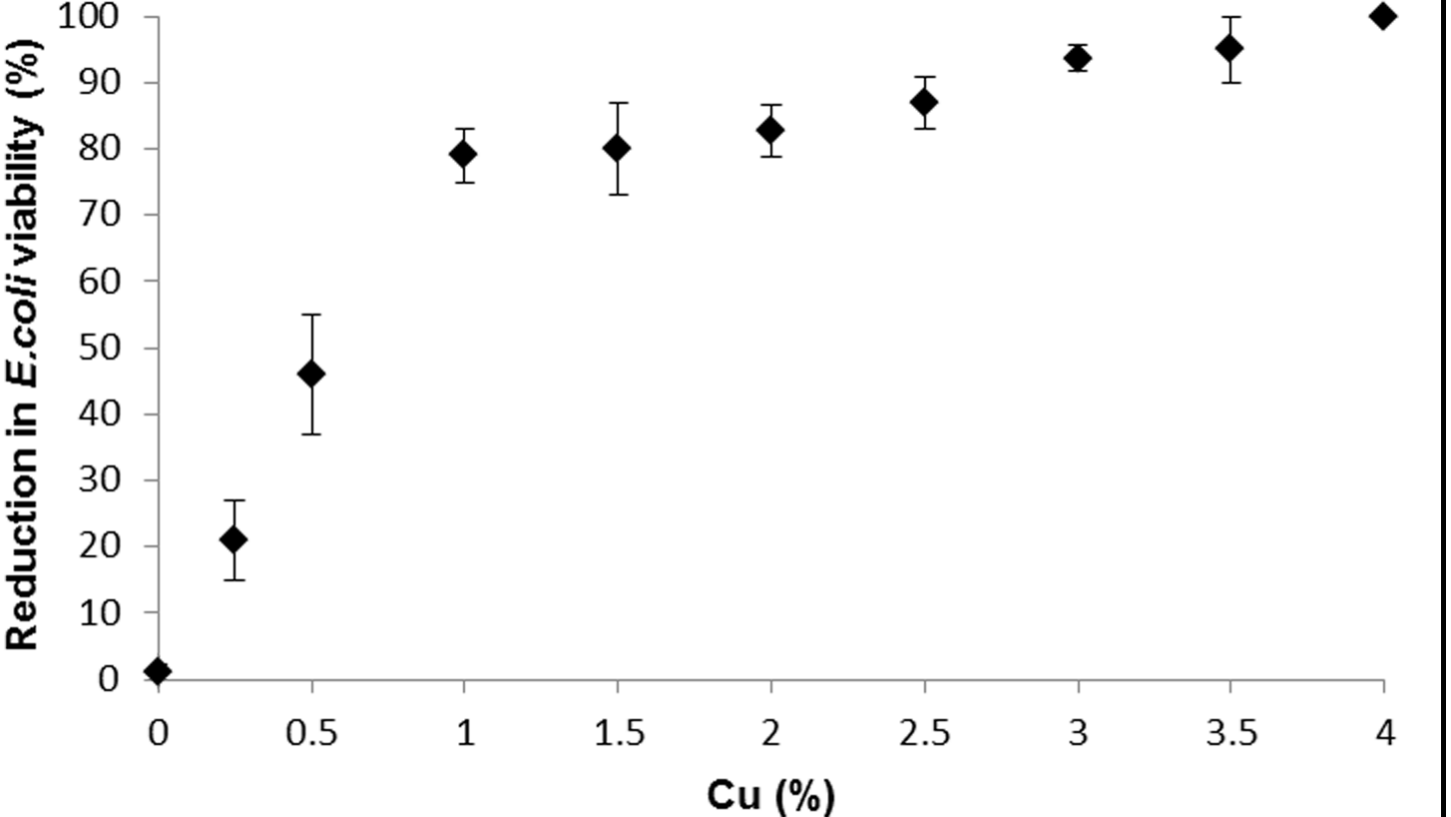
The response of *E. coli* ATCC 11775 to increasing copper concentrations in alginate beads after 90 minute exposure. Bars denote standard errors.

The 4% copper bead treatment produced a rapid reduction in viability of 10^8^ *ml^−1^ E. coli* cells, with 87.41% and 99.97% of bacteria rendered non-viable after 30 and 90 minutes respectively (Figure 1). The other treatments resulted in a dose related reduction of *E. coli* viability, with between 79 and 97% of bacteria non-viable after 90 minutes (Figure 2). However the 4% Cu bead treatment produced the most rapid viability reduction compared to the other treatments, with 87% of cells non-viable after 30 minutes compared to between 47–48.2% for the other treatments. Indeed, there was an almost instantaneous 49.7% reduction in viability immediately upon exposure to the 4% Cu beads. There was no reduction in *E. coli* viability observed in samples exposed to alginate beads containing no copper, and 4% copper particles not associated with alginate, only reduced *E. coli* viability by 44.2% after 90 minutes treatment (Figure 1).

MICs for Cu in alginate compared to Cu not associated with alginate also differed, with a value of 0.065% w:v Cu in alginate (0.02 mg/ml final concentration), preventing visible growth in an overnight culture of *E. coli,* whilst a dose of 0.5 mg/ml Cu powder, not associated with alginate, was required to similarly prevent visible growth.

There was a linear dose response of increasing Cu concentrations within the beads to the viability of *E. coli* cells from 0 to 1% Cu (R^2^ = 0.993[Figure 2]). Just 21.6% of cells were non-viable after 90 minutes exposure to 0.25% Cu, and this figure increased to 46.1% following exposure to 0.5% Cu. At the 1% Cu treatment, 76.5% of cells were non-viable. Increases in Cu concentrations above 1% led to a slower rate of loss of *E. coli* viability from 81% achieved at 1.5% Cu dose to >99.999% at the 4% Cu dose (Figure 2).

### Effect of Bacterial Numbers on Copper Efficacy

**Figure 3:**
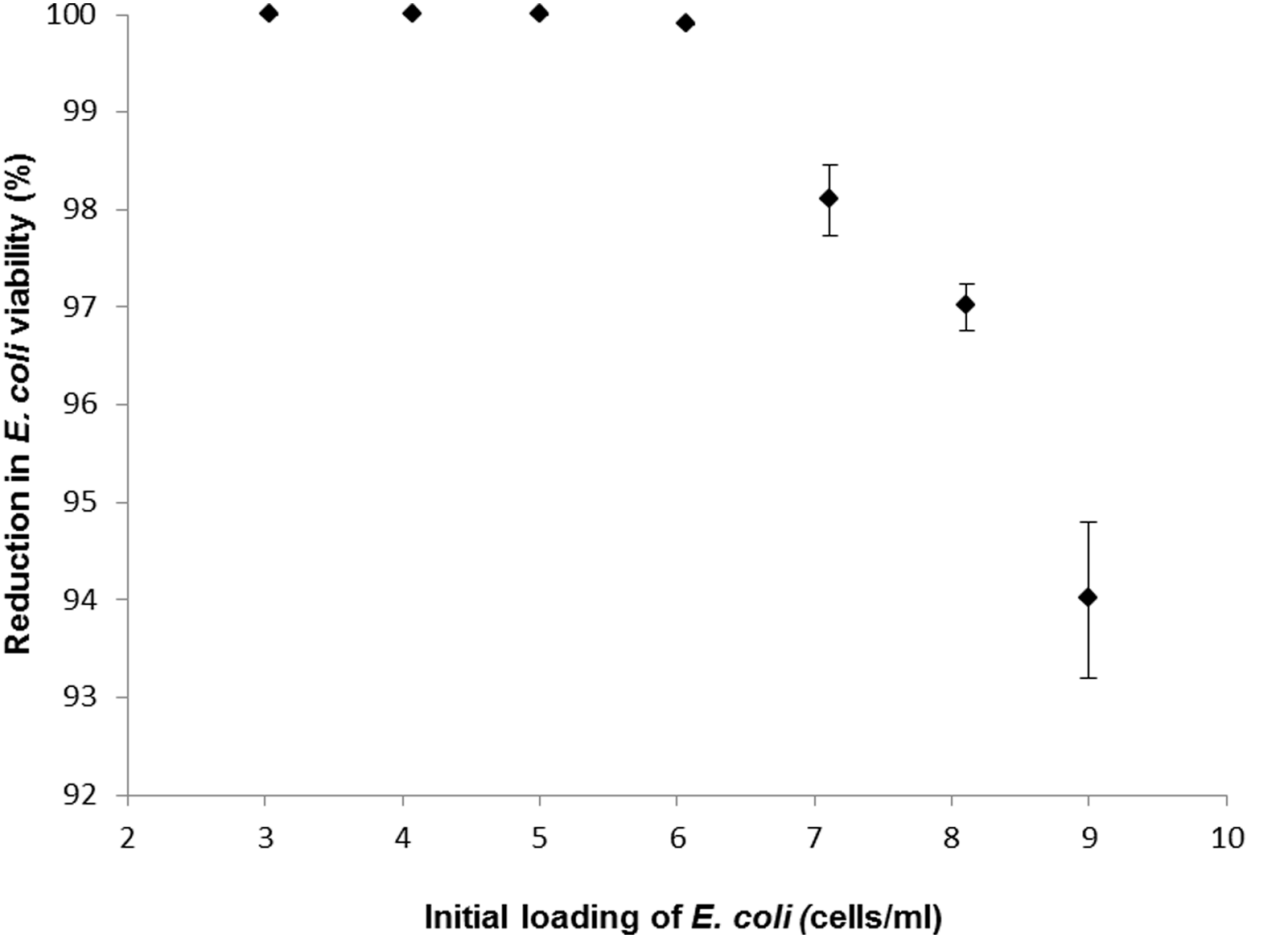
The relationship between increased bacterial loading and reduction in viability upon 90% dendritic copper in alginate beads. Bars denote standard errors.

At *E. coli* numbers up to 10^6^/ml more than 99.9% of cells were rendered non-viable after 90 minutes exposure (Figure 3), with no viable cells observed from an initial inoculum of 10^3^ cells/ml and 99.9997% of cells were non-viable at a level of 10^4^ cells/ml. However, this figure decreased as bacterial abundance increased above this figure, and at a loading of 10^8^ cells/ml, 88.2% of cells did not grow on MacConkey media and at 10^9^ cells/ml, this figure decreased further to 79.6% (Figure 3).

### The Relationship between Dissolved Oxygen (DO) Concentration in Media and the Reduction in *E. coli* Viability

**Figure 4:**
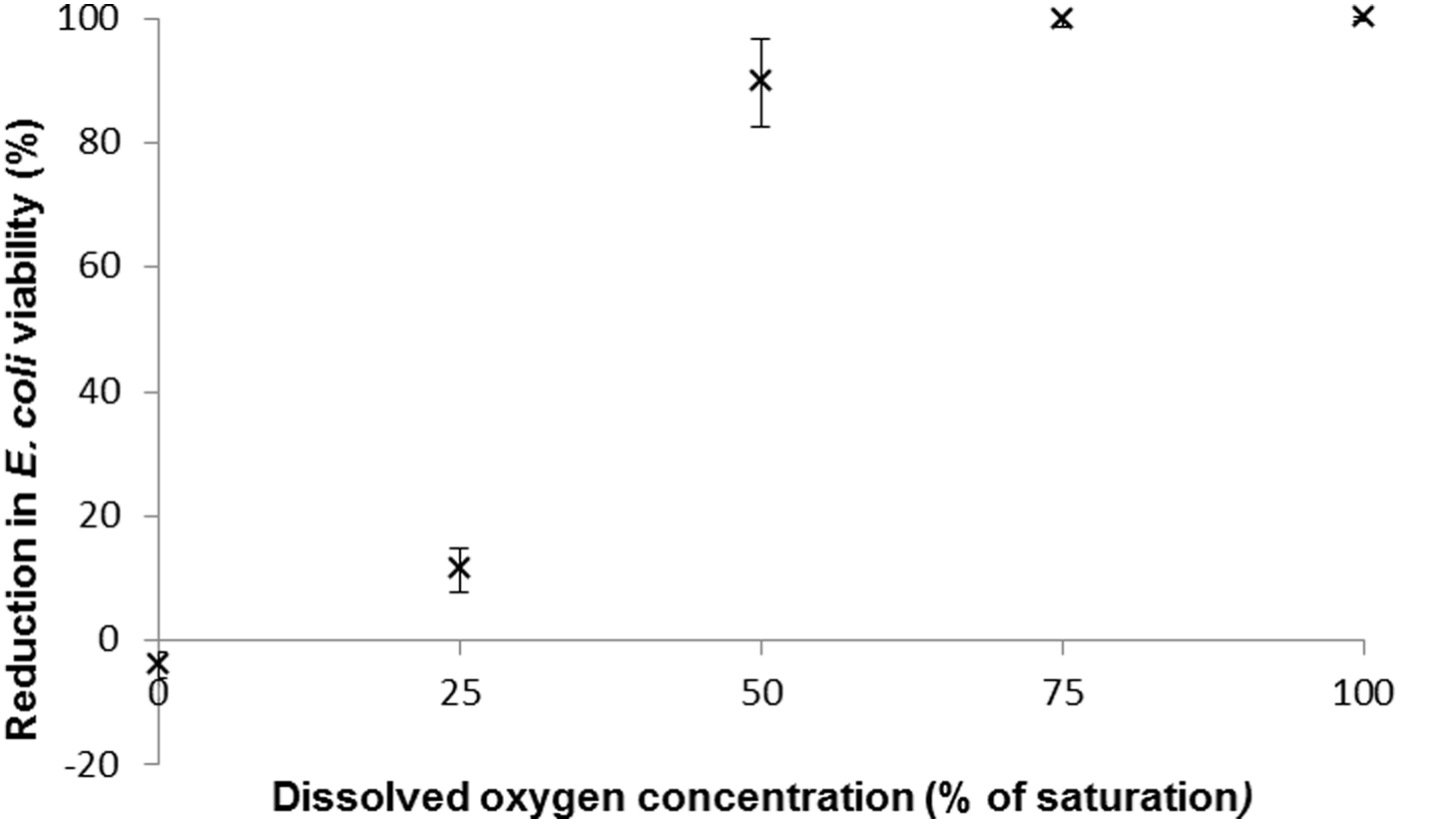
The relationship between dissolved oxygen concentration and *E. coli* ATCC 11775 viability using 4% Cu in alginate beads. The experiment was performed over 90****minutes at 37°C, with a bacterial loading of 10^6^ cells/ml. Bars denote standard errors.

**Figure 5:**
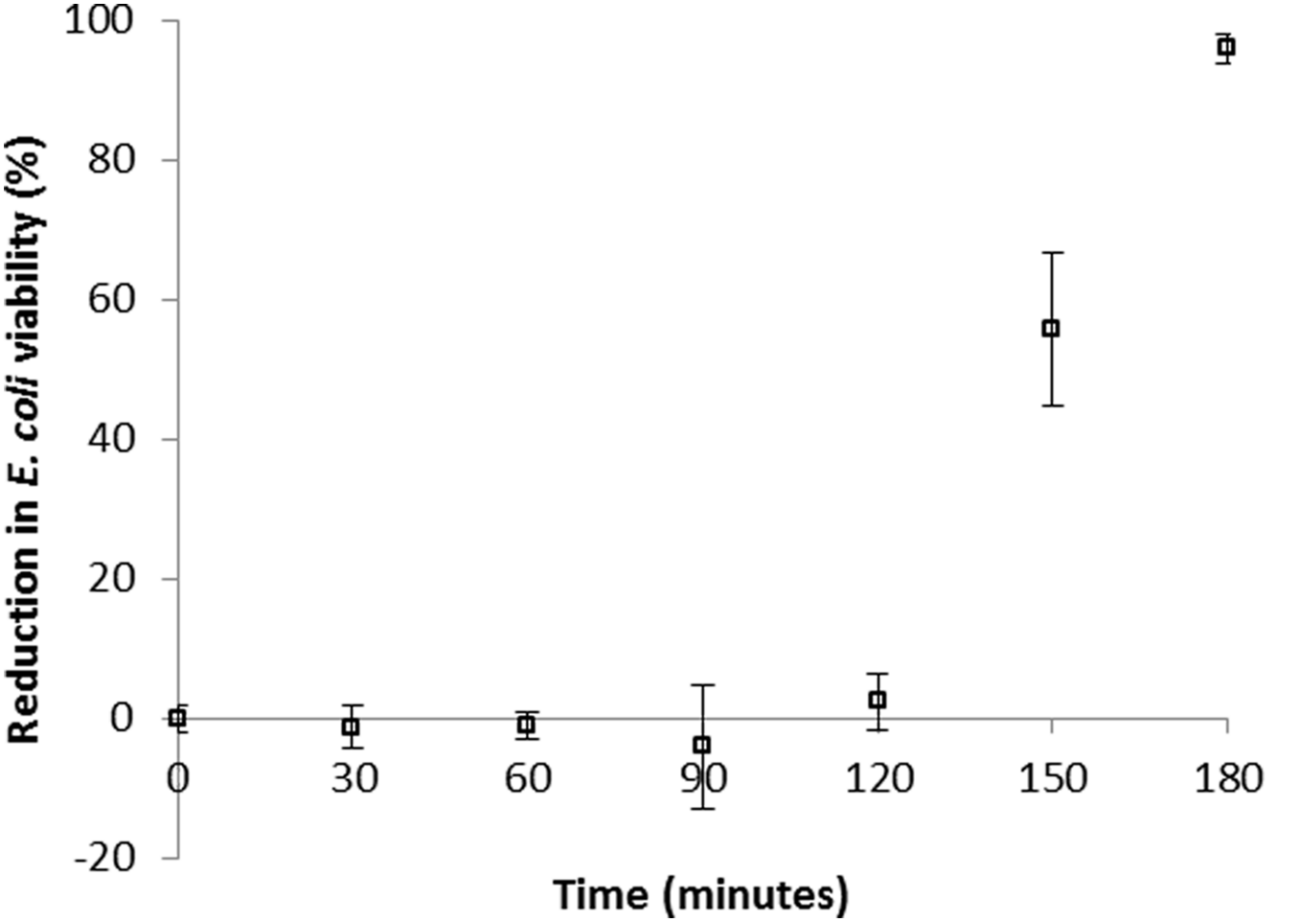
The viability of *E. coli* ATCC 11775 during treatment with 4% Cu (in alginate beads) under anoxic conditions (DO<0.1 mg/l). The experiment was performed at 37°C, with a bacterial loading of 10^6^ cells/ml.

When 10^6^ *E. coli* cells/ml were exposed to 4% Cu (in alginate beads) under anoxic conditions, no loss in viability was observed in the bacteria after 90 minutes exposure (Figure 4). Indeed an average 4.01% increase in viable bacteria was observed indicating that bacterial cell division occurred in the presence of bactericidal levels of copper, under low oxygen tensions. Once DO concentrations increased above 50% of saturation bacterial viability was rapidly reduced, and at 75 and 100% saturation, 99.7 and 99.997% respectively, of bacteria were no longer viable on MacConkey media (Figure 4). However, upon exposure for 180 minutes to Cu, *E. coli* cell viability began to decrease in the anoxic conditions and 96% of cells were no longer able to grow on agar plates (Figure 5).

### Quantification of Cu^2+^ Leaching and Absorption by Alginate Beads

The leaching of Cu^2+^, from dendritic Cu encapsulated in the Cu-Alginate beads, was below the limit of detection 48 hours post bead production (data not shown), despite minor leaching occurring in the initial 12 hours. The maximal leaching occurred between 8 and 9 hours post production and was measured at 0.4 mg/l at a rate of 0.2 mg/l/hr, however the Cu^2+^ concentration in the media subsequently decreased to below detection limits from 12 hours onwards, accompanied by a blue discolouration on the bead surface. The Cu^2+^ content of the media then remained unchanged and below the limit of detection until the termination of the experiment (48 hours).

### Electron Microscopy of *E. coli* and Alginate Beads

**Figure 6:**
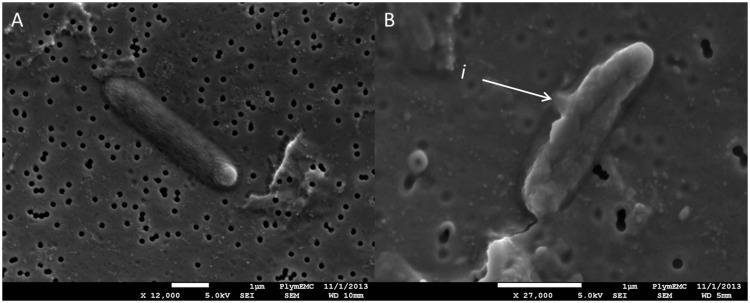
SEM of *E. coli* cells A: before and B: 45 minutes post treatment with Cu-containing alginate beads. *i* indicates disruption to cell membrane with associated loss in cellular integrity.

Morphological differences were observed between the 4% copper-alginate bead treated and untreated cells (Figure 6), with obvious membrane disruption observed in *E. coli* treated with copper after 90 minutes of exposure (Figure 6*i*). No morphologically intact cells were observed in the treated sample, whilst no membrane disruption was seen in any of the cells of the untreated sample. The copper-treated cells displayed clear blebbing, presumably due to the escape of intracellular material, which was not observed in either the untreated or time zero of the treated samples.

**Figure 7:**
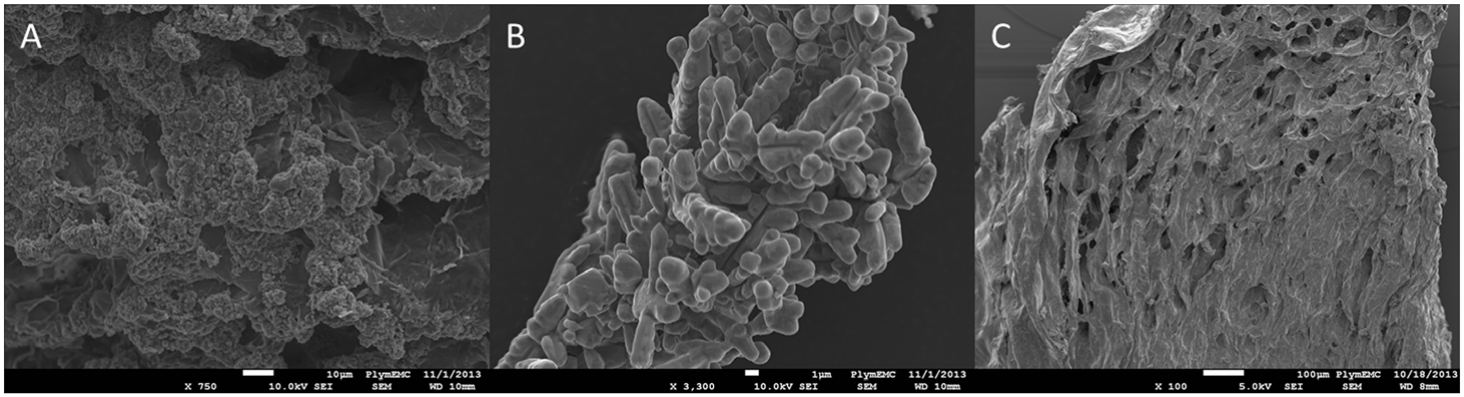
SEM of internal surface of alginate bead structure. A: Internal bead surface at x 750 magnification of dendritic copper particles visible, B: Internal bead surface at x 3300 times magnification of dendritic copper particle protruding from alginate matrix and C: A cross section of the Cu alginate bead.

The SEM visualisation of the alginate beads indicated a smooth, plated exterior, exhibiting a honey combed interior structure of the beads (Figure 7c). The dendritic copper particles are embedded in the layered alginate shells (Figure 7a), and the presence of copper was confirmed by x-ray analysis of the particles within the beads. Also, copper was detected evenly distributed throughout the bead interior, and this was thought to represent the Cu^2+^ ion produced from the oxidation of the elemental copper present. The dendritic structure of the copper could clearly be observed within the alginate matrix (Figure 7b).

## Discussion

During this study, the efficacy of alginate beads containing copper for the destruction of *E. coli* in an artificial media was established. The presence of dendritic Cu particles alone, did not exhibit the same degree of antimicrobial properties as the Cu bound within the alginate matrix. The synergistic effect between copper and alginate could be due to the stability provided by the alginate matrix in terms of shape and size of Cu. Dispersed or weakly aggregated metallic particles in suspension have a more variable internal structure and different reactivity and rates of surface reactions than strongly aggregated nanoparticles [13]. Subsequent changes in the physicochemical properties may alter the interaction of Cu particles with bacteria, thus affecting the antimicrobial activity. The immobilization of colloidal metal nanoparticles therefore prevents aggregation and provides positional stability to the nanoparticles on a surface, or in a structure, thus coordinating the interaction of Cu, bacteria and other functional groups present [17], [18].

The rapid kill rate was affected by low oxygen tensions in the media, supporting the observations of Baker *et al* that the initial damage caused to microbial cells is by the Cu^2+^ species and is oxidative in nature [19]. In anaerobic conditions the predominant copper species is Cu^+^ and the Cu(I)-translocating P-type ATPase responsible for regulating Cu homeostasis under such conditions is Cu^+^ specific [20]. However, under aerobic conditions the Cu^2+^ ion predominates and in most aqueous conditions this ion will be strongly bound to organic components. Indeed, Cu^2+^ is used as a catalyst in waste water treatment systems to oxidise organic waste [21], and such reactions are capable of producing reactive oxygen species through multiple pathways such as the Fenton-type reaction, which are highly toxic to many bacterial cells [22], [23]. Thus the 700 mg/l dissolved organic carbon present in the test media could provide an additive toxic effect through the production of ROS, thus providing interesting prospects for the use of this technique in the rapid deactivation of bacteria in recalcitrant waste waters with high organic loading, such as those found in many parts of the world that lack modern integrated sewerage treatment systems.

The electron micrographs of treated *E. coli* cells (Figure 6) indicated that cell membrane integrity is rapidly affected by Cu and the leakage of intracellular material can clearly be observed and this corresponds to the finding of Santo *et al* that the initial copper-induced cellular damage is membrane associated with consequent reduction in cellular osmotic stability [3]. An antimicrobial effect under anaerobic conditions was observed 2 hours post treatment, which suggests that the varying findings of different researchers, as to the exact nature of Cu-induced microbial lysis, may be due to different mechanisms, dependent on the redox status of the environment.

As the primary antimicrobial activity of Cu observed during this study appears oxidative in nature, the delay in observed lysis of *E. coli* under anoxic conditions may be due to a switch in microbial metabolism from respiratory to fermentative. Indeed Fung *et al* found that *E. coli* detoxification efflux mechanisms were more effective at removing Cu^+^ from the cell under anaerobic conditions than Cu^2+^ under aerobic [24]. However, under anaerobic conditions the turnover of Fe-S cluster enzymes appears to be especially sensitive to Cu-induced toxicity in *E. coli*. However, this toxicity was induced by Cu^+^ not Cu^2+^ as for aerobically-cultured organisms [24].

During this study the antimicrobial effect of micro as opposed to nano Cu particles in an alginate matrix is indicated for the first time. This is especially important at a time of increased interest in the microbial properties of Cu in the fields of medicine, public health and waste water treatment. The use of dendritic micro copper has advantages in terms of both cost and ease of manufacture over nanoparticles, for example dendritic copper sells for around $8–11 per kilo, whilst 50 nm Cu particles cost around $1200–1500 per kilo. This study suggests that Cu microparticles embedded within an alginate matrix have a strong potential for use as an antimicrobial in a wide range of clinical and industrial environments.
